# Aluminum Extractions by the Alkali Method Directly from Alkali-Acid (NaOH-HCl) Chemical Deashing of Coals

**DOI:** 10.3390/ma18153661

**Published:** 2025-08-04

**Authors:** Lijun Zhao

**Affiliations:** Advanced Materials Research Center, National Institute of Clean-and-Low-Carbon Energy, Future Science City, Changping District, Beijing 102211, China; zhaolijun@nicenergy.com

**Keywords:** aluminum extraction, alkali method, deashing solutions, coals

## Abstract

An advanced alkali-acid (NaOH-HCl) chemical method was used to deash aluminum-rich coals (ARCs) with a high ash content of 27.47 wt% to achieve a low ash content of 0.46 wt%. In the deashing process, aluminum in the coal ashes was dissolved in both alkali solutions and acid solutions. The deashing alkali solutions with dissolved coal ashes were regenerated by adding CaO, and the resulting precipitates were added with sodium bicarbonate for aluminum extraction. High temperatures increased aluminum extraction, and excessive sodium bicarbonate addition decreased aluminum extraction. The deashing acid solutions were concentrated by evaporation, and silica gels formed during the process. The obtained mixtures were calcinated at 350 °C for the decomposition of aluminum chlorides, and soaked with water at 60 °C to remove the soluble chlorides. For the insoluble oxides after soaking, diluted alkali solutions were used to extract the aluminum at 90 °C, and aluminum extraction failed due to the formation of albite in the presence of sodium, aluminum and silicon elements as proved by XRD and SEM/EDS. When silica gels were separated by pressure filtering, aluminum extraction greatly increased. Aluminum extractions were accordingly made in the form of sodium aluminate from the deashing solutions of coals, which could be advantageous for sandy alumina production.

## 1. Introduction

Aluminum is the third richest element in the crust of Earth, and bauxite is the most used mineral for alumina extraction. The Bayer process has been widely used to dissolve aluminum from bauxite by alkali solutions at high temperatures, and precipitate aluminum hydroxide by decreasing the temperatures, which can be further decomposed to sandy alumina by calcination [1]. For low-grade bauxite, a sintering method has been used to extract aluminum by adding lime at high temperatures, and leaching later with alkali solutions [1,2]. Moreover, because bauxite is not distributed evenly globally, for countries with limited bauxite, many nonbauxite minerals have also been considered, and aluminum has been extracted from clays [2,3], nepheline [4], chlorite [5], fly ashes [1,6,7], incense stick ash [8], and coals [9,10,11]. With aluminum-rich coals (ARCs), both alumina and ultra-clean coals can be obtained after deashing the coals [9,10,11].

In addition to alkali extractions in the form of sodium aluminate, acid extractions in the form of aluminum salts have also been developed for nonbauxite minerals, such as hydrochloric acid leaching [1]. Alkali extractions, which are typical of the Bayer process, can produce sandy alumina by the calcination of aluminum hydroxide with a weight loss of ~35% [12], which has been generally used by the modern electrolytic aluminum industry [1]. By comparison, alumina production from hydrochloric acid leaching would necessitate the calcination of aluminum chloride hexahydrate (ACH). Because the weight loss for ACH can be ~80% during calcination at high temperatures [13,14], alumina from ACH would essentially be pulverized. The resulting floury alumina by the calcination of ACH has been tested with poor physical properties, such as bulk density, repose angle and specific surface, which are very critical for the modern electrolytic aluminum industry [12,13]. To address the problem, ACH was calcinated at low temperatures to produce amorphous alumina with high activity, and similar processes like the Bayer process can be accordingly applied [15]. In our previous work [11], the sulfuric acid method was used for aluminum extraction from the deashing acid and alkali solutions of coals. For the aluminum-rich precipitates obtained by adding lime in the deashing alkali solutions [10], diluted solutions of sulfuric acid were added to dissolve the aluminum for combination with the deashing acid solutions. Highly concentrated sulfuric acid was used to precipitate aluminum sulfate octadecahydrate (ASO) from the combined acid solutions [10,16], and alumina was obtained by two-step calcination of ASO. There can be similar disadvantages for this method [11,13].

In addition to the sulfuric acid method [10], the alkali method was further developed in this work to extract aluminum from the deashing acid and alkali solutions of coals. In particular, for the deashing alkali solutions, lime was added to precipitate aluminum in the form of calcium aluminate (CA) hydrates [17], which can be used to extract aluminum by the addition of diluted solutions of sodium bicarbonate [18]. For the deashing acid solutions, evaporations were applied for the separation of silica [19] and collection of mixed salts. The mixed salts were calcinated and washed to remove the soluble chlorides, and diluted alkali solutions were used for aluminum extraction from the insoluble oxides. By comparison with the acid method, the alkali method developed in this work can make the aluminum in coals dissolved in alkali solutions, enabling the precipitation of aluminum hydroxide for decomposition to sandy alumina with desired properties.

## 2. Materials and Methods

Chemical deashing was applied to aluminum-rich coals (ARCs), and the deashing alkali and acid solutions were prepared with dissolved aluminum. Different strategies were adopted to extract aluminum, and the process is characterized in this work.

### 2.1. Preparation of the Deashing Alkali and Acid Solutions of Coals

An advanced alkali-acid (NaOH-HCl) chemical method was used to deash coals for preparation of the deashing alkali and acid solutions [9,10]. The aluminum-rich coals (ARCs) with a high ash content of 27.47 wt% were collected from the Province of Inner Mongolia of P. R. China, as given in Table 1. The ARC was crushed and passed through a sieve with a 1 mm mesh, and an alkali-to-coal ratio of 1.50 was used for the alkali treatment with little water at a temperature of 175 °C for 180 min. After the reaction, water was added to dilute the alkali-treated product for filtering, with 2–3 washes. The deashing alkali solutions were prepared with 10.19 g/kg, 2.49 g/kg and 179.68 g/kg of alumina (Al_2_O_3_), silica (SiO_2_) and Na_2_O, respectively. The ash content of dried coal cake after the alkali treatment was 27.42 wt%, as given in Table 1, similar to the raw coals. Alumina and silica in the ashes of raw coals were partially dissolved in the deashing alkali solutions, and the rest occurred with the included Na_2_O as the ashes of alkali-treated coals.

Subsequently, the alkali-treated coals were soaked by the diluted hydrochloric acid solutions of ~10 wt% with an acid-to-coal ratio of 1.5, and kept at 60 °C for 60 min. After that, the deashing acid solutions were prepared by filtering, and the filter cakes after 2–3 washes were ultra-clean coals (acid-treated coals) with a low ash of 0.46 wt%. Because carbon cannot be digested during the alkali-acid (NaOH-HCl) chemical deashing of coals [9,10], the adjusted ash contents can be obtained by normalization to the carbon contents. By using the adjusted ash contents and ash chemical compositions in Table 1, calculations showed that ~44.6 wt% and ~55.0 wt% of aluminum would be dissolved in the deashing alkali and acid solutions, and aluminum left in ultra-clean coals was ~0.4 wt%.

### 2.2. Aluminum Extractions from the Deashing Alkali and Acid Solutions of Coals

Similar to the previous procedures [10], the deashing alkali solutions of coals were regenerated by adding CaO with stirring at 75 °C for 60 min, and the aluminum-rich precipitates were obtained by filtering. By slightly varying the addition amount, sodium bicarbonate has been previously used for aluminum extractions from calcium aluminate hydrate in the temperate range of 100–160 °C, and higher temperatures were found to favor aluminum extraction [18]. Therefore, a slurry was made in this work by pulping 4.0 g aluminum-rich precipitates with 40 mL water, sodium bicarbonate was added and aluminum extractions were made at the temperatures of 95 °C and 160 °C for 180 min in sealed 200 mL Teflon bottles, which were fixed in the rotary homogeneous reactor (KLJX-8A, Keli Chemical Equipment Co., Ltd., Yanntai, China). After that, the reaction mixtures were cooled and filtered, the filter liquors were transferred to 50 mL plastic bottles for ICP-AES analysis, and the filter cakes after drying were collected for XRD characterization. By varying the addition amount of sodium bicarbonate in a much wider range than previously used [18], the process of aluminum extraction was investigated in this work. In addition, the aluminum extraction rate can be defined according to Formula (1), as the percentage of the aluminum in filter liquors with respect to the aluminum in aluminum-rich precipitates. Aluminum in filter liquors was analyzed twice by ICP-AES, and the average was used for the calculation of extraction rate. Aluminum in aluminum-rich precipitates was analyzed and quantified by XRF. Due to unstable filter liquors, especially for aluminum extractions with high addition amounts of sodium bicarbonate, some precipitates could be quickly identified on the walls of 50 mL plastic bottles, which were not included in the calculation of aluminum extraction rates according to Formula (1).

Al extraction rate = (Al_filter liquors_/Al_aluminum-rich precipitates_) × 100% (1)

The deashing acid solutions of coals were concentrated by the rotary evaporator (Chemtron Strike 300, Perugia, Italy). Silica gels formed during the evaporation [19], and two routes were accordingly investigated for aluminum extraction. For the first route, silica gels were not separated. The mixtures after evaporation were calcinated at the temperature of 350 °C for 180 min, and the calcined products were soaked with water at the temperature of 60 °C for 60 min to remove the soluble chlorides after filtering, and the remaining insoluble oxides were treated for aluminum extraction by the diluted alkali solutions of ~10 wt% at the temperature of 90 °C for 60 min. For the second route, silica gels were separated, and similar procedures were applied for aluminum extraction.

### 2.3. Chemicals and Instrumental Analysis

Alkali (NaOH) and NaHCO_3_ of analytic grade were used, and HCl of 37 wt% was diluted as required by deionized water. Lime (CaO) was prepared by calcination of Ca(OH)_2_ of analytic grade at 850 °C for 180 min. A proximate analyzer (5E-MAG6700II, CKIC, Changsha, China) was used for measurement of coal ash content. XRF (ZSX Primus II, Rigaku, Tokyo, Japan) was used for elemental analysis (Be-U), and XRD (D8 Advance, Bruker, Berlin, Germany) was used for phase characterizations of solid powders. ICP-AES (Spectro Arcos, Spectro, Kleve, Germany) was used for elemental analysis of the deashing solutions of coals. SEM with EDS accessory (Nova NanoSEM 450, FEI, Brno, Czech Republic) was used for elemental analysis (Be-U) and mapping spatial distributions of specific elements.

## 3. Results and Discussions

Due to its amphoteric property, aluminum was dissolved in both alkali solutions and acid solutions in the deashing of coals, as indicated in Table 1. Therefore, different strategies should be developed to extract aluminum from the deashing alkali and acid solutions. For the alkali solutions, sodium bicarbonate was used to extract aluminum [18]. For the acid solutions, two routes were investigated, i.e., with or without silica separation.

### 3.1. Aluminum Extraction from the Deashing Alkali Solutions of Coals

According to the previous results, deashing alkali solutions could be added with lime for regeneration. In proportion to alumina [10], lime was increased to 1.33 g/50 mL in this work. After addition of CaO with stirring at 75 °C for 60 min, the concentrations of alumina and silica were 0.68 g/kg and 0.24 g/kg, very close to 0.77 g/kg and 0.18 g/kg in the previous work [10]. Due to more alumina and silica in the deashing alkali solutions in this work, the removal rates for alumina and silica were increased to 93.7% and 90.2%.

**Figure 1:**
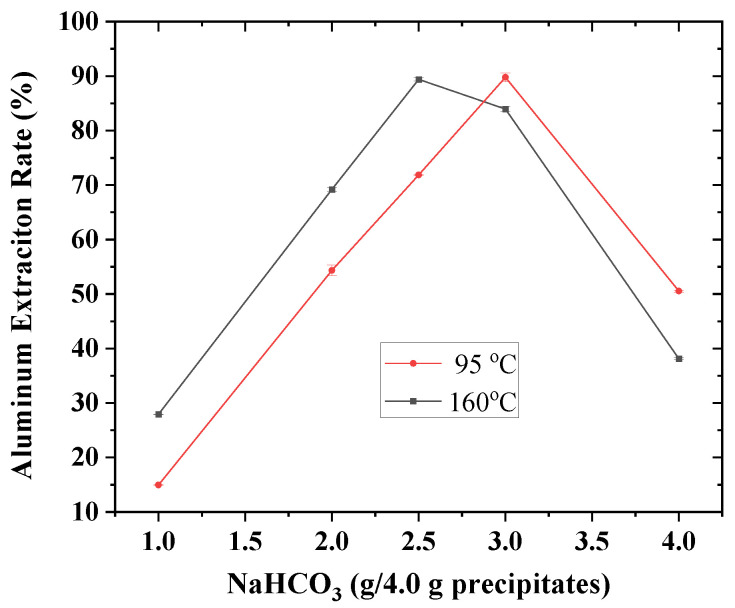
Effects of temperature and sodium bicarbonate addition on aluminum extraction rate (with error bars) of aluminum-rich precipitates obtained from the alkali regeneration by adding lime.

With the addition of lime for alkali regeneration, the aluminum-rich precipitates were analyzed by XRF, and the results are given in Table 2. Aluminum extractions could be made by reacting sodium bicarbonate with calcium aluminate hydrate for the formation of sodium aluminate, calcium carbonate and sodium hydroxide [18]. According to Figure 1, for the reactions at 160 °C, the highest extraction rate was above 90% at the sodium bicarbonate addition of 2.5 g/4.0 g precipitates, and with adding more of the reagent, the extraction rate decreased to below 40%. For the reactions at 95 °C, the extraction rate steadily increased from below 20% to over 90% with increasing the reagent addition from 1.0 to 3.0 g/4.0 g precipitates. For the experiments at 95 °C at the reagent addition of 4.0 g/4.0 g precipitates, the aluminum extraction descended to about 50%. Therefore, higher temperatures could be beneficial to aluminum extractions. Though necessary for aluminum extractions, sodium bicarbonate could decrease the extraction rates if added in excess [12,18].

**Figure 2:**
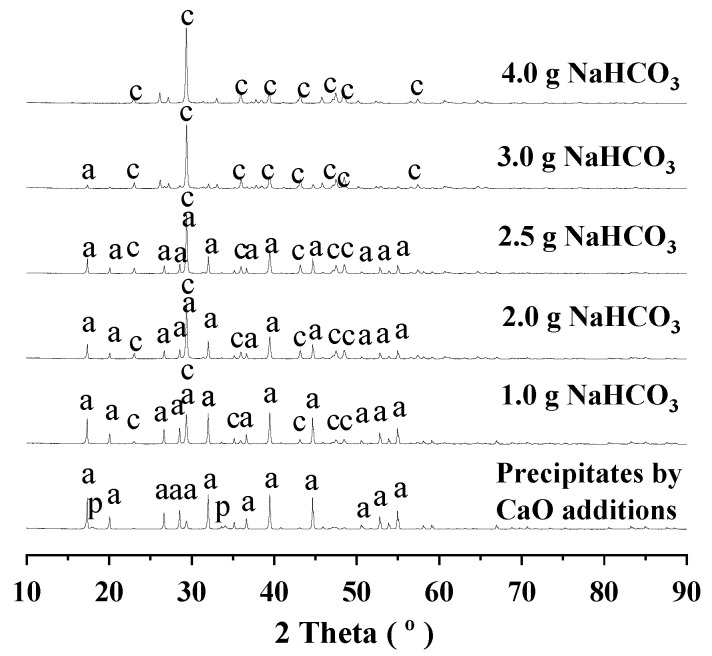
XRD diagram of the residues after the aluminum extraction at 95 °C. Note: a for CA hydrate (PDF 77-0240), p for portlandite (PDF 76-0571), c for calcite (PDF 86-0174) in the diagram.

**Figure 3:**
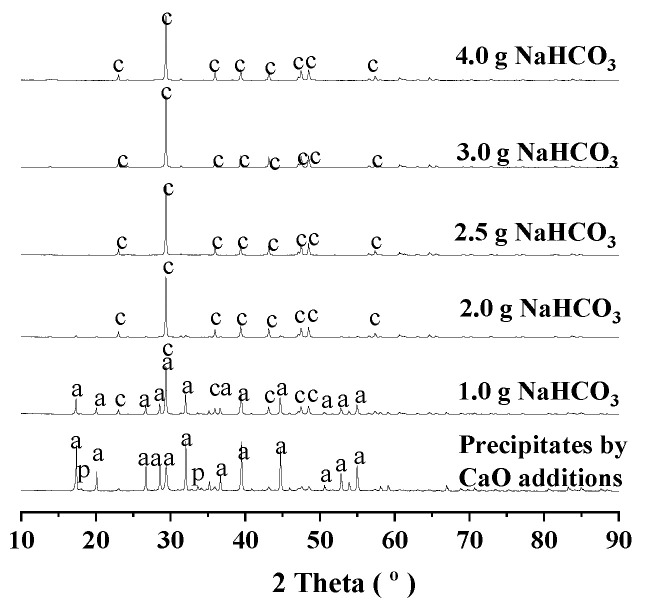
XRD diagram of the residues after the aluminum extraction at 160 °C. Note: a for CA hydrate (PDF 77-0240), p for portlandite (PDF 76-0571), c for calcite (PDF 86-0174) in the diagram.

For the aluminum extractions by the addition of sodium bicarbonate, the residues were collected for XRD investigations, and the results were given in Figure 2 and Figure 3. For the aluminum-rich precipitates obtained from the alkali regeneration by the addition of lime, the phases were identified as CA hydrate (PDF 77-0240), portlandite (PDF 76-0571). For the reactions at 95 °C, with increasing the addition of sodium bicarbonate, portlandite (PDF 76-0571) disappeared very rapidly, and CA hydrate (PDF 77-0240) gradually weakened with growing calcite (PDF 86-0174). For the reactions at 160 °C, with increasing the addition of sodium bicarbonate, both portlandite (PDF 76-0571) and CA hydrate (PDF 77-0240) disappeared very rapidly with growing calcite (PDF 86-0174).

**Figure 4:**
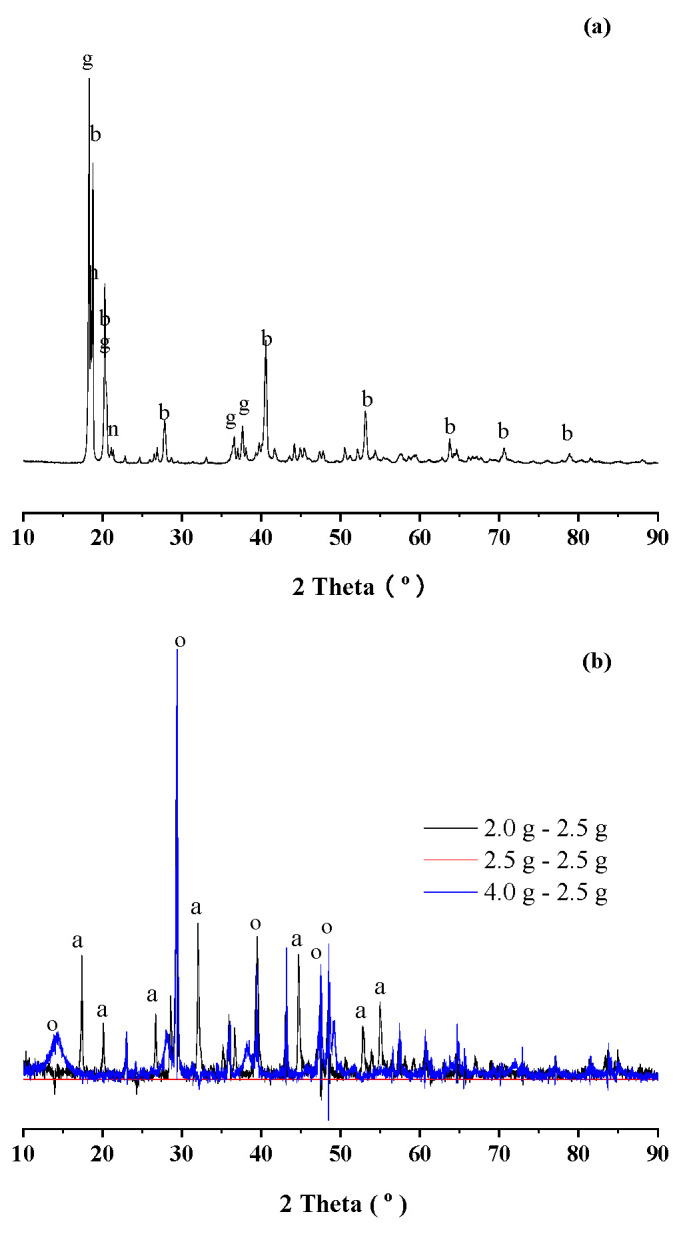
(**a**) XRD diagram of new precipitates collected from the walls of plastic bottles for the aluminum extraction at 95 °C with the sodium bicarbonate addition of 4.0 g/4.0 g precipitates. (**b**) XRD difference spectra for the aluminum extractions at 160 °C for the sodium bicarbonate additions of 2.0 and 4.0 g/4.0 g precipitates, with respect to the sodium bicarbonate addition of 2.5 g/4.0 g precipitates. Note: g for gibbsite (PDF 74-1775), b for bayerite (PDF 74-1119), n for nordstrandite (PDF 85-1049), a for CA hydrate (PDF 77-0240), o for boehmite (PDF 74-1895) in the diagram.

For the reaction at 95 °C with the sodium bicarbonate additions of 4.0 g/4.0 g precipitates, some new precipitates were collected from the walls of plastic bottles, for investigation by XRD in Figure 4a. Gibbsite (PDF 74-1775) and bayerite (PDF 74-1119) were identified as the dominant components, in addition to nordstrandite (PDF 85-1049). For the XRD diagram in Figure 3 for the reactions at 160 °C, by normalizing the peak at around 30°, the XRD difference spectra in Figure 4b were made for the sodium bicarbonate additions of 2.0 and 4.0 g/4.0 g precipitates, with respect to the sodium bicarbonate addition of 2.5 g/4.0 g precipitates with the highest aluminum extraction rate. With the addition of sodium bicarbonate of 2.0 g/4.0 g precipitates, CA hydrate (PDF 77-0240) could be clearly observed, indicating that sodium bicarbonate could not be enough for completing the reaction with CA hydrate (PDF 77-0240). With the addition of sodium bicarbonate of 4.0 g/4.0 g precipitates, boehmite (PDF 74-1895) could be identified in the XRD difference spectra. By comparison with gibbsite, bayerite and nordstrandite, boehmite would be more stable at high temperatures [20]. Therefore, with sodium bicarbonate being added in excess for the aluminum extractions at 160 °C, boehmite could be formed.

### 3.2. Route One for Aluminum Extractions from the Deashing Acid Solutions of Coals

According to Table 1, the ash contents were 27.42 wt% and 0.46 wt% for alkali-treated coals and ultra-clean coals (acid-treated coals); therefore, the ashes in alkali-treated coals would be dissolved almost totally in the deashing acid solutions for aluminum extractions.

**Figure 5:**
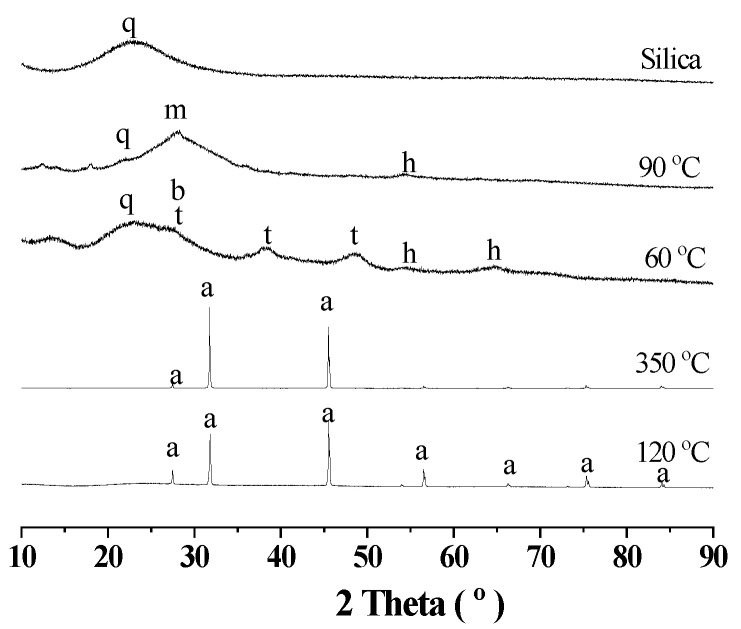
XRD diagram for the process of aluminum extraction without silica separation. The deashing acid solutions were dried at 120 °C and calcinated at 350 °C. The calcinated products were then soaked with water at 60 °C, and treated with diluted alkali solutions at 90 °C for aluminum extraction. Dried silica gels were compared. Note: a for halite NaCl (PDF 78-0751), b for bayerite (PDF 83-2256), m for albite (PDF 84-0982), t for anatase (PDF 78-2486), h for hematite (PDF 87-1166), q for silicon oxide (PDF 29-0085) in the diagram.

In Route One, silica gels were not separated during the evaporation of the deashing acid solutions. The concentrated acid solutions were then dried at 120 °C for the mixed salts, which were mostly NaCl according to Figure 5. Because aluminum chloride hexahydrate (ACH) could be decomposed at 350 °C [1,14], the mixed salts were accordingly calcinated to obtain the active alumina for aluminum extraction by diluted alkali solutions.

After the calcination at 350 °C according to Figure 5, the mixed salts were dominated by NaCl. When the products after calcination were soaked with water to remove soluble salts like NaCl in Table 3, the residues could be identified with silicon oxide (PDF 29-0085), bayerite (PDF 83-2256), anatase (PDF 78-2486) and hematite (PDF 87-1166) in Figure 5. When the residues were further treated by diluted alkali solutions, bayerite as the active alumina could be easily dissolved. However, according to Table 3 and Figure 5, albite (PDF 84-0982) was formed with the inclusion of large amounts of alkali. Therefore, bayerite could have been dissolved with silicon oxide (PDF 29-0085) by diluted alkali solutions in the first step, and albite was formed in the second step. The chemical compositions in Table 3 for the dried powders of filter cakes at 60 °C demonstrated that NaCl was effectively removed by soaking with water. The dried powders for filter cakes at 90 °C were rich with alumina and silica, indicating the failure of aluminum extraction.

**Figure 6:**
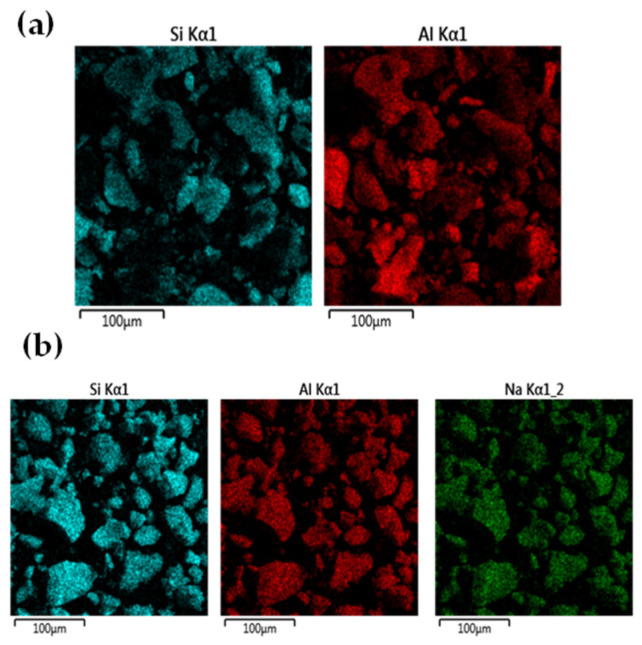
Role of silicon in aluminum extraction, highlighted by the SEM/EDS images of the spatial distributions of elements. (**a**) SEM/EDS images of the same region for dried cakes soaked by water at 60 °C after calcination at 350 °C, and (**b**) SEM/EDS images of the same region for dried cakes treated by diluted alkali solutions at 90 °C, for aluminum extraction without silica separation.

In Figure 6, the role of silicon in Route One can be highlighted by the SEM/EDS images of spatial distributions of silicon, aluminum and sodium elements. In Figure 6a, for the dried cakes soaked with water at 60 °C after calcination at 350 °C, the same region was investigated by SEM/EDS, and the blue patterns contributed by silicon element can be observed to separate from the red patterns contributed by aluminum element. This should be attributed to the formation of silica gels during evaporation of deashing acid solutions [10,19], prior to calcination and water soaking. In Figure 6b, for the dried cakes treated by diluted alkali solutions at 90 °C, the same region was also investigated by SEM/EDS; the blue patterns contributed by silicon element can be observed to strikingly resemble the red and green patterns contributed by aluminum and sodium elements. This should be attributed to the formation of albite after treatment by diluted alkali solutions.

### 3.3. Route Two for Aluminum Extractions from the Deashing Acid Solutions of Coals

In Route One for aluminum extraction from the deashing acid solutions, silica gels have been found to prevent aluminum extraction. Therefore, to extract aluminum from the deashing acid solutions, silica gels should be separated in advance [10,19].

**Figure 7:**
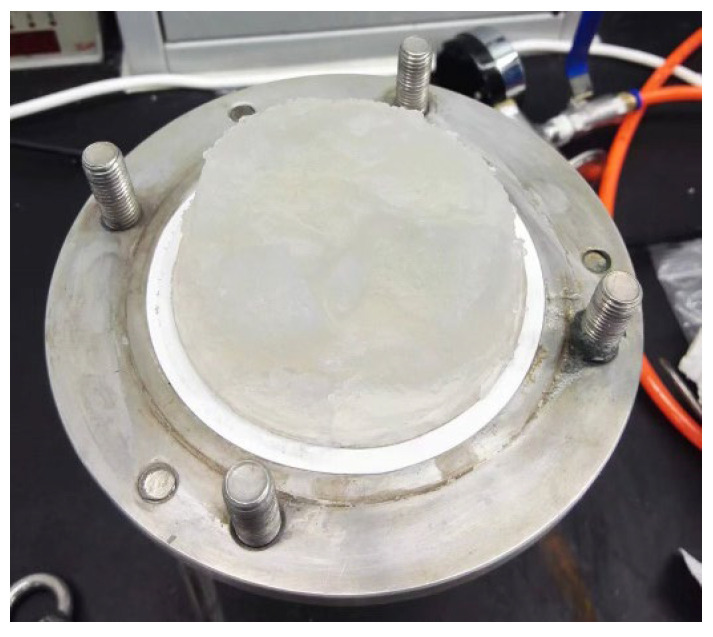
Silica gels filtered from the deashing acid solutions of coals.

In this work, the deashing acid solutions were concentrated by evaporation, so that silica gels could be formed and filtered for separation. Instead of vacuum filtering, pressure filtering was applied for the silica separation in Figure 7. The chemical compositions of dried silica gels are given in Table 4, and the purity of silica was 96.4 wt%. The filter liquors with silica separation were further dried at 120 °C and calcinated at 350 °C, and the chemical compositions are given in Table 5. Silica was absent or negligible in the process flows when silica separation was carried out in advance. By soaking with water at 60 °C after calcination at 350 °C, alumina could account for 81.5 wt% of dried cakes. When diluted alkali solutions were used, almost all aluminum was successfully extracted, by referring to the insoluble TiO_2_ or Fe_2_O_3_ in dried cakes at 90 °C. The chemical compositions of dried powders of filter liquors in Table 5 should indicate the removal of NaCl at 60 °C by soaking with water, and aluminum extraction by diluted alkali solutions at 90 °C.

**Figure 8:**
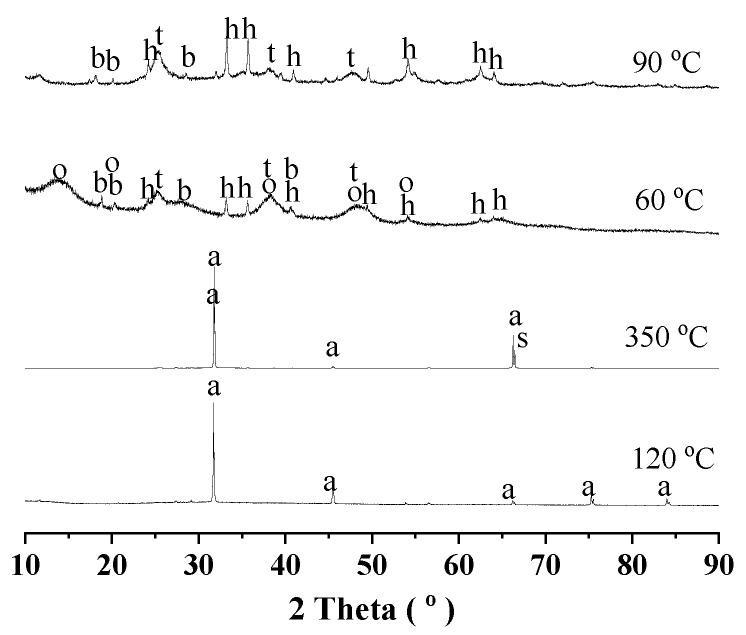
XRD diagram for the process of aluminum extraction with silica separation. Deashing acid solutions were dried at 120 °C and calcinated at 350 °C. The calcinated products were then soaked with water at 60 °C, and treated with dilute alkali solutions at 90 °C for aluminum extraction. Note: a for halite (PDF 78-0751), h for hematite (PDF 87-1166), b for bayerite (PDF 83-2256), t for anatase (PDF 78-2486), s for ssigma alumina (PDF 80-1385), o for boehmite (PDF 88-2112) in diagram.

The residues from Route Two with silica separation for aluminum extraction were investigated by XRD, and the results are given in Figure 8. For filtrate liquors with silica separation, the phases after drying at 120 °C and calcination at 350 °C remained almost the same as those without silica separation, except for one weak peak of sigma alumina (PDF 80-1385) appearing after calcination at 350 °C. By soaking with water at 60 °C, bayerite (PDF 83-2256) and boehmite (PDF 88-2112) occurred, and some hematite (PDF 87-1166) and anatase (PDF 78-2486) also appeared in the dried cakes. After the aluminum extraction by diluted alkali solutions at 90 °C, the dried cakes were mostly hematite and anatase, which were in good agreement with the chemical compositions in Table 5.

### 3.4. Defining Aluminum Extractions in the Context of Deashing Auminum-Rich Coals (ARCs)

**Figure 9:**
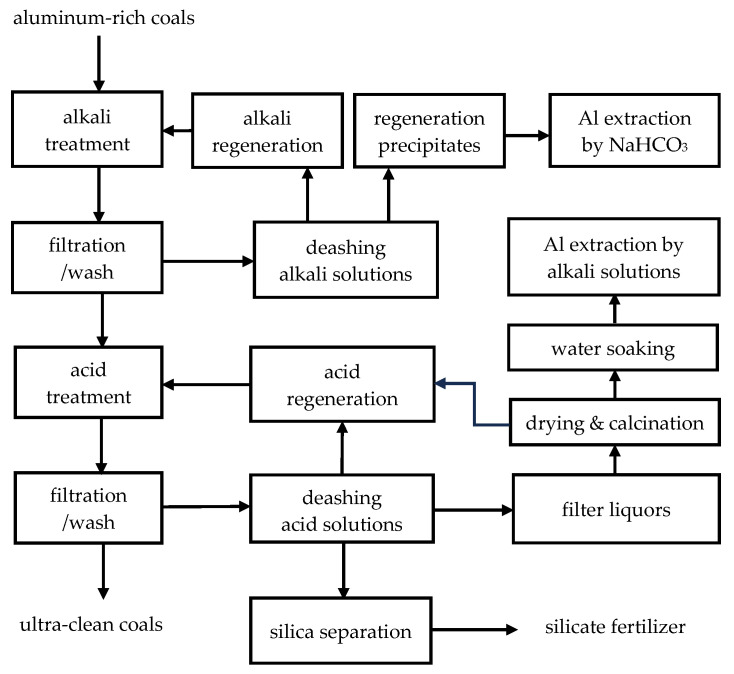
Schematic diagram of deashing the aluminum-rich coals (ARCs), with ultra-clean coals, silicon and aluminum products.

According to the investigations carried out in this work, sodium bicarbonate was successfully used to extract aluminum from the deashing alkali solutions in Section 3.1. For the deashing acid solutions, Route One without silica separation failed for aluminum extraction in Section 3.2, and Route Two with silica separation proved to be very effective for aluminum extraction in Section 3.3. Therefore, the two procedures developed in Section 3.1 and Section 3.3 should be integrated for aluminum extractions from the deashing alkali and acid solutions. As a result, for alkali-acid (NaOH-HCl) chemical deashing of aluminum-rich coals (ARCs), aluminum extractions can be defined in Figure 9.

According to Figure 9, aluminum-rich coals (ARCs) were treated by alkali and acid in sequence for the production of ultra-clean coals. The deashing alkali solutions were regenerated by the addition of CaO, for reuse in the alkali treatment of coals, and the precipitates were treated by sodium bicarbonate for aluminum extraction. The deashing acid solutions were evaporated for reuse in the acid treatment of coals. Silica gels formed during the evaporation and were separated by pressure filtering for the preparation of silicate fertilizers [10,19]. The filter liquors after silica separations were dried and calcinated at low temperatures for the formation of active alumina. The released acid gas could be absorbed for reuse in the acid treatment of alkali-treated coals. The calcinated products were soaked with water for removing the soluble chlorides, and finally the diluted alkali solutions were used to extract aluminum from the insoluble oxides after water soaking. By comparison with the acid extractions, the aluminum extractions in the form of sodium aluminate should be advantageous for the preparation of sandy alumina [21].

Following the general scheme in Figure 9, deashing ARC could yield ultra-clean coals with many advanced applications [22,23,24,25]. To eliminate secondary pollution and reduce the deashing cost, the deashing alkalic and acid solutions should be treated to regenerate the chemicals and develop silicon and aluminum products. The process design has been awarded to Aohua Engineering Co. LTD, and a preliminary analysis showed that the deashing cost could be ~853 RMB/ton of coals in northwestern provinces of China. The overall economy should consider the outputs of deashing coals, such as the ultra-clean coals, silicon and aluminum products, which can vary depending on conditions.

## 4. Conclusions

In this work, aluminum extractions were successfully made in the form of sodium aluminate. By using the advanced alkali-acid (NaOH-HCl) deashing method [9,10], ultra-clean coals with a low ash content of 0.46 wt% were prepared. For the deashing alkali solutions, lime (CaO) was added for the regeneration of alkali with high alumina and silica removal rates of 93.7% and 90.2%, resulting accordingly in the aluminum-rich precipitates. Sodium bicarbonate was successfully used to extract aluminum from the precipitates, and extraction conditions were investigated for the reaction temperatures and addition amounts of the reagent. High temperature increased the aluminum extraction, and excessive sodium bicarbonate decreased the aluminum extraction by destabilizing the extraction solutions. For the deashing acid solutions, silica gels formed during the evaporation, and two routes were investigated, i.e., with or without silica separation. The filter liquors were dried and calcinated at 350 °C for the formation of active alumina. After that, the soluble chlorides were removed from calcinated products by water soaking at 60 °C, and for the insoluble oxides, diluted alkali solutions were used to extract aluminum at 90 °C. Following the same procedures, aluminum extractions with silica separation were greatly improved, by comparison with the poor aluminum extraction without silica separation. The combined alkali solutions with the dissolved aluminum from the deashing alkali and acid solutions of coals can be treated by seeding and carbonation, to precipitate sandy aluminum hydroxide for further decomposition to sandy alumina [21].

This work also demonstrates an ecological approach for coal deashing, adhering to the principle of best material utilization. The improved aluminum extractions in this work should be integrated in the process of deashing coals, which can help to eliminate secondary pollution and reduce the deashing cost, together with silicon products.

## Figures and Tables

**Table 1:** The ash contents (wt%) by proximate analyzer, and the ash chemical compositions (wt%) in terms of oxides by XRF, for the raw coals, alkali-treated coals and acid-treated coals.

Sample	Ash wt%	Na_2_O wt%	MgO wt%	Al_2_O_3_ wt%	SiO_2_ wt%	SO_3_ wt%	K_2_O wt%	CaO wt%	TiO_2_ wt%	Fe_2_O_3_ wt%
Raw Coals	27.47	1.2	0.4	47.0	33.3	2.7	0.6	5.1	3.8	5.0
Alkali-treated Coals	27.42	26.2	0.4	26.1	28.7	4.3	0.5	5.0	3.3	4.7
Acid-treated Coals	0.46	1.9	0.4	13.7	48.9	3.7	0.2	10.3	10.0	7.1

**Table 2:** The chemical compositions (wt%) in terms of oxides by XRF for aluminum-rich precipitates obtained by the addition of lime (1.33 g/50 mL) in the deashing alkali solutions.

Na_2_O wt%	MgO wt%	Al_2_O_3_ wt%	SiO_2_ wt%	P_2_O_5_ wt%	SO_3_ wt%	CaO wt%	Fe_2_O_3_ wt%
0.3	1.3	24.5	5.0	0.2	0.1	68.3	0.3

**Table 3:** The chemical compositions (wt%) in terms of oxides by SEM/EDS for the aluminum extraction from the deashing acid solutions without silica separations. The filter cakes were dried, and the filter liquors were evaporated and dried for measurements. Note: bdl for “below detection limit of 0.1 wt%”.

Route One	Na_2_O wt%	Al_2_O_3_ wt%	SiO_2_ wt%	SO_3_ wt%	Cl wt%	CaO wt%	TiO_2_ wt%	Fe_2_O_3_ wt%
350 °C salts calcination	29.0	20.8	15.4	0.4	30.9	1.7	1.0	1.0
60 °C filter cakes dried	0.3	45.5	46.7	0.8	0.3	1.3	2.8	2.4
90 °C filter cakes dried	18.1	32.5	42.2	bdl	bdl	1.5	3.1	2.7
60 °C filter liquors dried	44.5	0.5	bdl	0.4	52.0	2.6	bdl	bdl
90 °C filter liquors dried	74.3	19.1	6.7	bdl	bdl	bdl	bdl	bdl

**Table 4:** The chemical compositions (wt%) in terms of oxides by XRF of dried silica gels, which were separated from deashing acid solutions by pressure filtering.

Na_2_O wt%	Al_2_O_3_ wt%	SiO_2_ wt%	SO_3_ wt%	Cl wt%	K_2_O wt%	TiO_2_ wt%	Fe_2_O_3_ wt%
0.3	0.1	96.4	0.4	0.7	0.1	1.7	0.3

**Table 5:** The chemical compositions (wt%) in terms of oxides by SEM/EDS for aluminum extraction from the deashing acid solutions with silica separations by pressure filtering. The filter cakes were dried, and the filter liquors were evaporated and dried for measurements. Note: bdl for “below detection limit of 0.1 wt%”.

Route Two	Na_2_O wt%	Al_2_O_3_ wt%	SiO_2_ wt%	SO_3_ wt%	Cl wt%	CaO wt%	TiO_2_ wt%	Fe_2_O_3_ wt%
350 °C calcination	13.0	46.5	bdl	1.5	23.9	7.8	2.9	4.3
60 °C filter cakes dried	0.5	81.5	0.1	1.6	0.3	0.6	8.4	7.0
90 °C filter cakes dried	bdl	4.8	bdl	bdl	bdl	3.3	41.1	50.8
60 °C filter liquors dried	39.1	0.4	bdl	0.6	51.4	8.5	bdl	bdl
90 °C filter liquors dried	76.4	23.6	bdl	bdl	bdl	bdl	bdl	bdl

## Data Availability

The original contributions presented in the study are included in the article. Further inquiries can be directed to the corresponding author.
